# Supplementary material for: VPS13B is localized at the interface between Golgi cisternae and is a functional partner of FAM177A1
Source: J Cell Biol. 2024 Sep 27;223(12):e202311189. doi: 10.1083/jcb.202311189 (PMC11451052; doi:10.1083/jcb.202311189)
Supplement: Table S1 — shows a list of primers used in this study. [file JCB_202311189_TableS1.docx]

**Supplemental Tables**

**Table S1.** List of primers used in this study.

| *VPS13B* Guide 1 sequencing forward | CAAATGGGGAAAAATTAAGAACC |  |
| --- | --- | --- |
| *VPS13B* Guide 1 sequencing reverse | ACTTCCAGCCTGGGGATATT |  |
| *VPS13B* Guide 2 sequencing forward | TTCGGCCTGAGATAACGAAC |  |
| *VPS13B* Guide 2 sequencing reverse | TGTAAAATAAAGCCCCCTTGTT |  |
| *VPS13B N586Fragment-F* | TGCTTAaccggtccCAAACGAAAATCAAGAGGACCTATAACA |  |
| *VPS13B N586Fragment-R* | AATGCAGAGCTCgccaccATGCTGGAGTCATATGTAACT |  |
| *VPS13B N586 S560=>563D_F* | agtggcaaaggttccacaaatcaacaagacttttctgatgggaaagatgaagatttgggaacagttc |  |
| *VPS13B N586 S560=>563D_R* | gaactgttcccaaatcttcatctttcccatcagaaaagtcttgttgatttgtggaacctttgccact |  |
| *VPS13B LTD mut1 g-block seq (top strand)* | ATGCTCGAGTCTTACGTTACACCCATCCTCATGAGTTACGTAAATAGATACATCAAAAATCTGAAGCCTTCTGACTTACAGCTGAGTTTGTGGGGCGGCGACGTGGTCCTGTCGAAGCTAGAATTGAAGCTTGACGTGCTAGAACAGGAACTGAAACTCCCCTTCACCTTCTTGTCGGGCCACATCCACGAGAAGAGGATACATGTGCCATGGACCAAGCTGGGTAGCGAGCCTGTGGTCGAGACAATCAACACTATGGAATGTATCGAGAAGCTGAAGGATGGCATCCAAGACGACCACGAGAGCTGCGGCAGTAACTCTACCAACAGAAGCACAGCCGAGTCGACCAAGAGCTCTATCAAGCCACGGCGGATGCAGCAGGCCGCCCCCACTGACCCCGACCTCCCGCCTGGTTATGTACAGTCCCTGATCAGACGAGTGGTGAACAACGTGAACATCGTGCGGAACAACCTAATCCTGAAGTACGTGGAAGACGACATTGTTGAGTCTGTGAACATCACATCTGAGGAATGCTACACCGTGGGGGAACTGTGGGACCGGGCTTTCATGGACATAAGTGCCACAGACCTGGTGCTCCGCAAGGTGAGAAATTTCAGCGACTGCACCGTGTGTCTTGATAAGAGAAATGCCTCTGGCAAAATCGAGTTCTACCAAGATCCCCTCCTGTACAAGTGCTCTTTCAGAACTAGAGACCACTTCACCTACGAGAATTTGAACAGCAAAATGCCCTCAGTCATTAAGAAGCACACACTGGTGGAATCGCTAAAGCGGAGTATCACCGACCAACAATTACCTATGTTTATC |  |
| *LTD-mut1_Hifi_Fragment1-F* | CAATTACCTATGTTTATCAGGAT |  |
| *LTD-mut1_Hifi_Fragment1-R* | TTTGTGTCTTAGGCAGCTTG |  |
| *LTD-mut1_Hifi_Fragment2-F* | CAGCATCTTCCAGGCCAAGCT |  |
| *LTD-mut1_Hifi_Fragment2-R* | GAAGGCCTCTTCGTCCAGGGT |  |
| *LTD-mut1_Hifi_Fragment3-F* | GAGAGGTTGTGACCCTGGACG |  |
| *LTD-mut1_Hifi_Fragment3-R* | TGCAAAAGCCTAGGCCTCCAA |  |
| *LTD-mut1_Hifi_Fragment4-F* | GGAGGCTTTTTTGGAGGCCTA |  |
| *LTD-mut1_Hifi_Fragment4-R* | GGATGGGTGTAACGTAAGACT |  |
| *FAM177A1* sequencing forward | CACCGATATAGATGAGTAACGAAA |  |
| *FAM177A1* sequencing reverse | AAACCTTTCGTTACTCATCTATAT |  |
| *FAM177A1* Fragment1 Halo Forward | CTCGAGATTTCCGGCtgataaacccgctgatcagc |  |
| *FAM177A1* Fragment1 Halo Reverse | CATTCCACTTCCTCCtggtgggacagagactggat |  |
| *FAM177A1* Fragment2 Halo Forward | ccaGGAGGAAGTGGAATGGCAGAAATCGGTACTGGCTTT |  |
| *FAM177A1* Fragment2 Halo Reverse | ttatcaGCCGGAAATCTCGAGCGTCGACA |  |
| *FAM177A1*_Fragment1 Snap Forward | CTGGGTtgataaacccgctgatcagcctc |  |
| *FAM177A1* Fragment1 Snap Reverse | CATTCCACTTCCTCCtggtgggacagagactggat |  |
| *FAM177A1* Fragment2 Snap Forward | ccaGGAGGAAGTGGAATGGACAAAGACTGCGAAATGA |  |
| *FAM177A1*_Fragment2_Snap reverse | atcagcgggtttatcaACCCAGCCCAGGCTTG |  |
| *FAM177A1_helix1_gblock* | GCTAGCGCTACCGGACTCAGATCTCGAGGCCACCATGGGAGGATCATGGGGTCCCTACTTATGGTTTTACATGCTTCGGGCTGCTACATCAACTCTCTCAGTGTGTGACTTCCTTGGAGAGAAGATTGCATCTGTTGGTGGTTCACCGCGGGCCCGGGATCCACCGGTCGCCACCATG |  |
| *FAM177A1_helix2_gblock* | GCTACCGGACTCAGATCTCGAGGCCACCATGGGAGGATCACAATATGCCATTGATGAATATTATCGGATGAAGAAGGAGGAAGAAGAGGAAGAAGAGGAAAACAGGATGTCTGAAGAAGCAGAAAAACAATATCAACAGAATAAATTGCAGACTGATTCCATTGTTCAGGGTGGTTCACCGCGGGCCCGGGATCCACCGGTCGC |  |
| *FAM177A1_hairpin_gblock* | GCTAGCGCTACCGGACTCAGATCTCGAGGCCACCATGGGTGGTTCAAGGAGAGTCATCCACTTTGTTAGTGGTGAAACAATGGAAGGTGGTTCACCGCGGGCCCGGGATCCACCGGTCGCCACCATG |  |
| *FAM177A1_deltahelix1_sense* | AAAACTTACCTTGGGTATCAGCACCCCAAA |  |
| *FAM177A1_deltahelix1_antisense* | TGATACCCAAGGTAAGTTTTGTCGGATCAA |  |
| **Zebrafish study** |  |  |
| **Primers used in the qPCR and genotyping** | | **Sequence** |
| *vps13b* qPCR forward | CCGTCAGACACCACAGATCT |  |
| *vps13b* qPCR reverse | AGATTCACTGCAATGCCCTGA |  |
| *rpl13a* qPCR forward | TCTGGAGGACTGTAAGAGGTATGC |  |
| *rpl13a* qPCR reverse | AGACGCACAATCTTGAGAGCAG |  |
| *vps13b*_geno_F | GTTCCTGCCCTCCGACACAC |  |
| *vps13b*_geno_R | GCAGACACACCACCAGCGT |  |
|  |  |  |
