# Supplementary material for: VPS13B is localized at the interface between Golgi cisternae and is a functional partner of FAM177A1
Source: J Cell Biol. 2024 Sep 27;223(12):e202311189. doi: 10.1083/jcb.202311189 (PMC11451052; doi:10.1083/jcb.202311189)
Supplement: Table S2 — shows FLASH-PAINT Nanobody sequences. [file JCB_202311189_TableS2.docx]

**Table S2. FLASH-PAINT Nanobody Sequences**

| **Target** | **Nanobody** | **Sequence (3’ 🡪 5’)** |
| --- | --- | --- |
| VSP13b-GFP | Anti-GFP-Nanobody-A3 | NB - TT TCTTCATTAGCG |
| TGN46 | Anti-Rabbit-Nanobody-A15 | NB - TT ATAGTGATTGGA |
| GM130 | Anti-Rabbit-Nanobody- A39 | NB - TT TTATGTTCTGCT |
| GOLGA1_1 Golgin-97 | Anti-Rabbit-Nanobody-A8 | NB - TT ATGTTAATGGGT |
| GRASP65 | Anti-Rabbit-Nanobody-A38 | NB - TT ATTTAGTGTAGC |
| GOLGB1 Giantin | Anti-Rabbit-Nanobody-A20 | NB - TT ATATGATCTCCG |
| COPI (CMIA10) | Anti-Mouse-Nanobody-A27 | NB - TT AAAAAGTTCGAG |
