# Supplementary material for: VPS13B is localized at the interface between Golgi cisternae and is a functional partner of FAM177A1
Source: J Cell Biol. 2024 Sep 27;223(12):e202311189. doi: 10.1083/jcb.202311189 (PMC11451052; doi:10.1083/jcb.202311189)
Supplement: Table S3 — shows FLASH-PAINT Imager sequence. [file JCB_202311189_TableS3.docx]

**Table S3. FLASH-PAINT Imager Sequence**

| **Imager name** | **Sequence** | **3'-mod** |
| --- | --- | --- |
| R2-6nt | TGGTGG | Cy3B |
