# Supplementary material for: VPS13B is localized at the interface between Golgi cisternae and is a functional partner of FAM177A1
Source: J Cell Biol. 2024 Sep 27;223(12):e202311189. doi: 10.1083/jcb.202311189 (PMC11451052; doi:10.1083/jcb.202311189)
Supplement: Table S4 — shows FLASH-PAINT Adapter sequences. [file JCB_202311189_TableS4.docx]

**Table S4. FLASH-PAINT Adapter Sequences**

| **Adapter Name** | **Sequence (3’ 🡪 5’)** |
| --- | --- |
| A3-5xR2 | ACCACCACCACCACCACCA AA CGCTAATGAA |
| A15-5xR2 | ACCACCACCACCACCACCA AA TCCAATCACT |
| A39-5xR2 | ACCACCACCACCACCACCA AA AGCAGAACAT |
| A8-5xR2 | ACCACCACCACCACCACCA AA ACCCATTAAC |
| A38-5xR2 | ACCACCACCACCACCACCA AA GCTACACTAA |
| A20-5xR2 | ACCACCACCACCACCACCA AA CGGAGATCAT |
| A27-5xR2 | ACCACCACCACCACCACCA AA CTCGAACTTT |
