# Supplementary material for: VPS13B is localized at the interface between Golgi cisternae and is a functional partner of FAM177A1
Source: J Cell Biol. 2024 Sep 27;223(12):e202311189. doi: 10.1083/jcb.202311189 (PMC11451052; doi:10.1083/jcb.202311189)
Supplement: Table S5 — shows FLASH-PAINT Eraser sequences. [file JCB_202311189_TableS5.docx]

**Table S5. FLASH-PAINT Eraser Sequences**

| **Eraser Name** | **Sequence (3’ 🡪 5’)** |
| --- | --- |
| E3-5xR2 | TTCATTAGCG TT TGG |
| E15-5xR2 | AGTGATTGGA TT TGG |
| E39-5xR2 | ATGTTCTGCT TT TGG |
| E8-5xR2 | GTTAATGGGT TT TGG |
| E38-5xR2 | TTAGTGTAGC TT TGG |
| E20-5xR2 | ATGATCTCCG TT TGG |
| E27-5xR2 | AAAGTTCGAG TT TGG |
