# Supplementary material for: VPS13B is localized at the interface between Golgi cisternae and is a functional partner of FAM177A1
Source: J Cell Biol. 2024 Sep 27;223(12):e202311189. doi: 10.1083/jcb.202311189 (PMC11451052; doi:10.1083/jcb.202311189)
Supplement: Table S6 — shows FLASH-PAINT Adapter and Imager concentrations. [file JCB_202311189_TableS6.docx]

**Table S6. FLASH-PAINT Adapter and Imager Concentrations**

| **# Round** | Protein | Adapter – Concentration | Imager – Concentration |
| --- | --- | --- | --- |
| Round 1 | VPS13b | A3-5xR2 – 20 nM | R2 – 200pM |
| Round 2 | GM130 | A15-5xR2 – 20 nM | R2 – 500 pM |
| Round 3 | TGN46 | A39-5xR2 – 20 nM | R2 – 200 pM |
| Round 4 | COPI | A8-5xR2 – 20 nM | R2 – 500 pM |
| Round 5 | Golgin97 | A38-5xR2 – 20 nM | R2 – 500 pM |
| Round 6 | Giantin | A20-5xR2 – 20 nM | R2 – 500 pM |
| Round 7 | Grasp65 | A27-5xR2 – 20 nM | R2 – 300 pM |
