# Supplementary figures and images for: VPS13B is localized at the interface between Golgi cisternae and is a functional partner of FAM177A1
Source: J Cell Biol. 2024 Sep 27;223(12):e202311189. doi: 10.1083/jcb.202311189 (PMC11451052; doi:10.1083/jcb.202311189)

**Table S7. Violin Plot of Data represented in Fig. 1H**

**
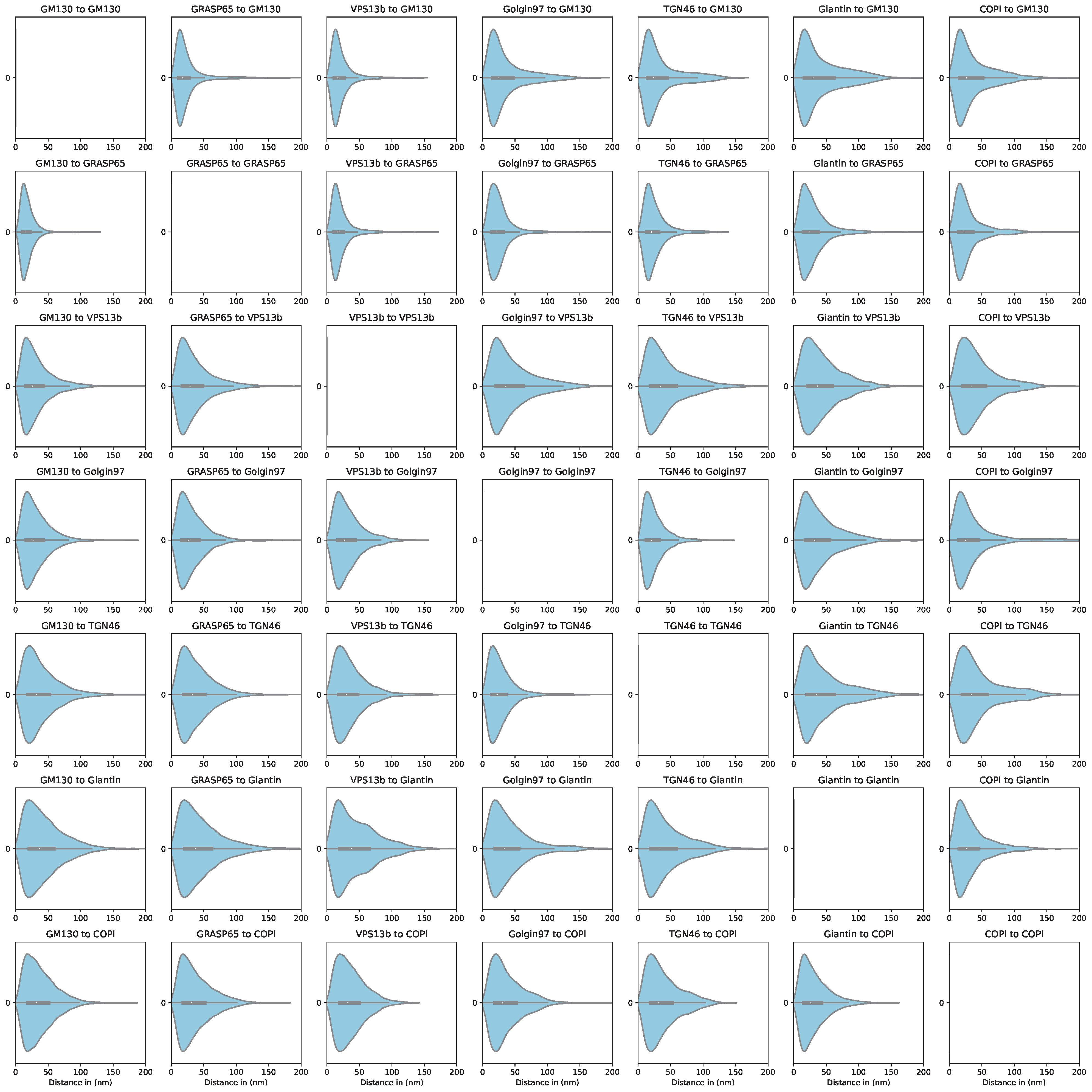
**

Supplement: Table S7 — shows a violin plot of data represented in Fig. 1 H. [file JCB_202311189_TableS7.docx]

**Table S9. Violin plot of data presented in Fig. 2F**

**
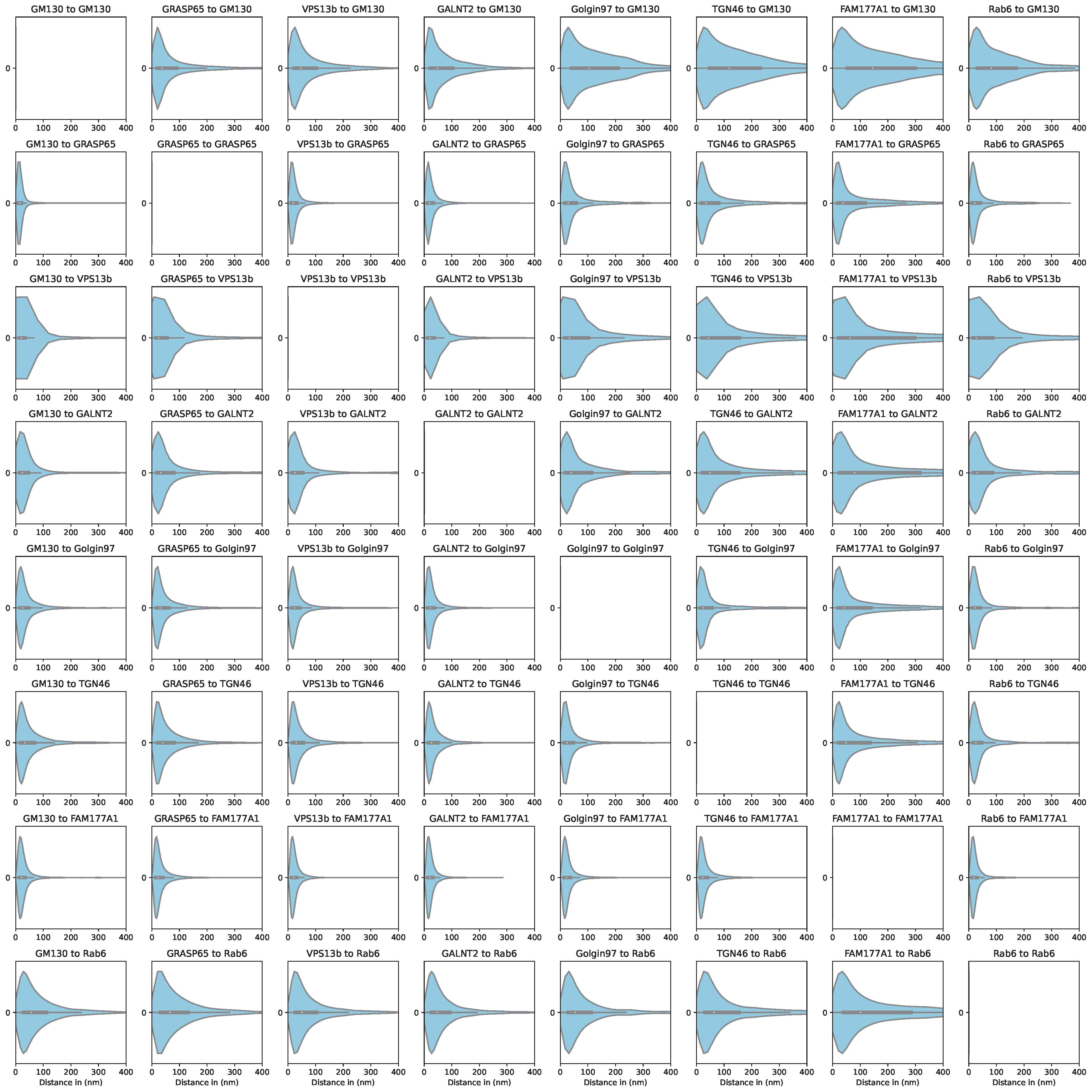
**

Supplement: Table S9 — shows a violin plot of data presented in Fig. 2 F. [file JCB_202311189_TableS9.docx]

Source Data Figure 2C

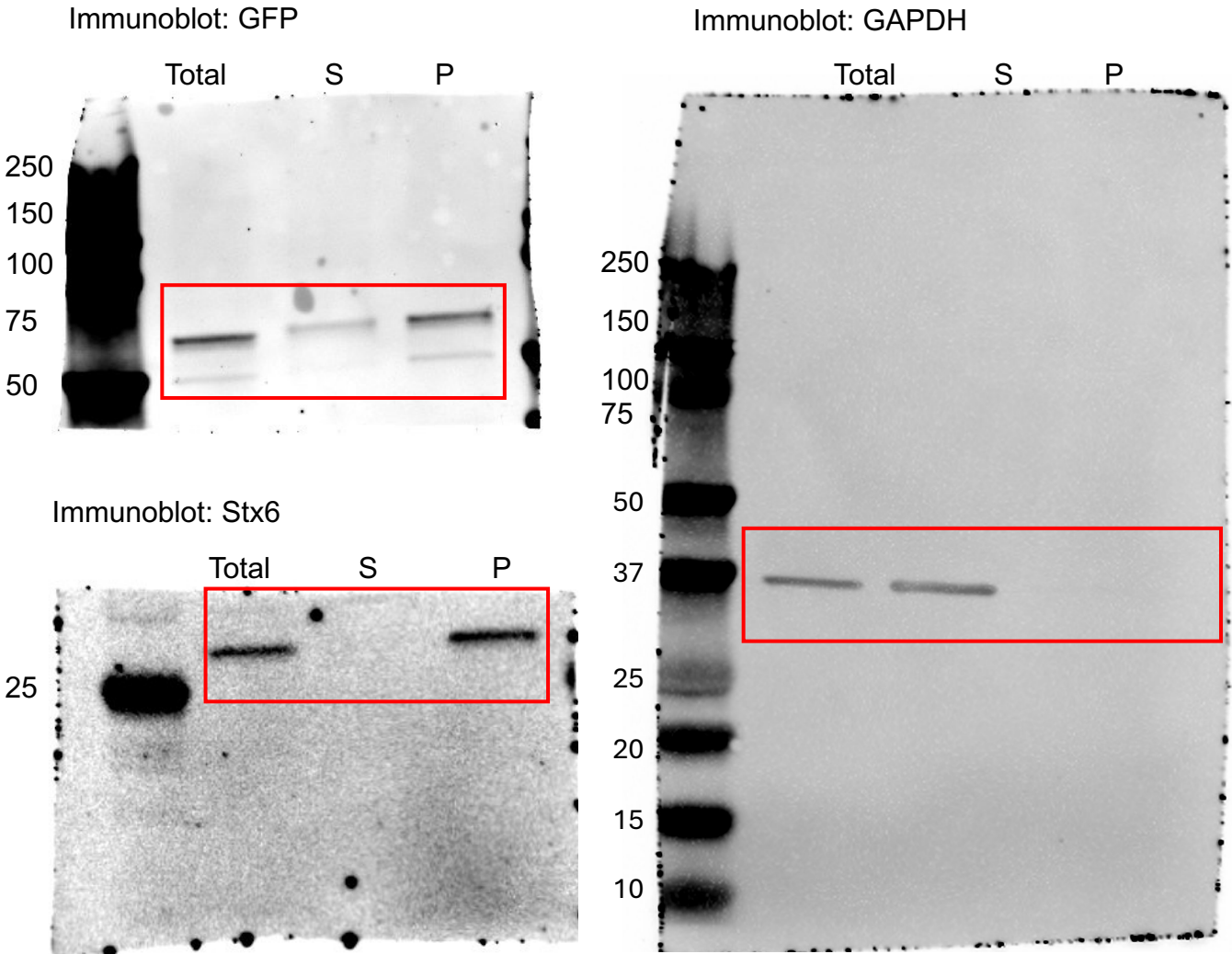

Supplement: SourceData F2 — is the source file for Fig. 2. [file JCB_202311189_SourceDataF2.pdf]
