# Supplementary material for: VPS13B is localized at the interface between Golgi cisternae and is a functional partner of FAM177A1
Source: J Cell Biol. 2024 Sep 27;223(12):e202311189. doi: 10.1083/jcb.202311189 (PMC11451052; doi:10.1083/jcb.202311189)
Supplement: Table S8 — shows a localization precision table in relation to Fig. 1 H. [file JCB_202311189_TableS8.docx]

**Table S8. Localization precision table in relation to Fig. 1H**

| **Target** | **Localization precision (nm)** |
| --- | --- |
| VPS13B | 16.8 |
| GM130 | 15.7 |
| TGN46 | 14.8 |
| beta-COP | 16.1 |
| Golgin-97 | 18.9 |
| Giantin | 14 |
| GRASP65 | 15.8 |
