# Supplementary material for: VPS13B is localized at the interface between Golgi cisternae and is a functional partner of FAM177A1
Source: J Cell Biol. 2024 Sep 27;223(12):e202311189. doi: 10.1083/jcb.202311189 (PMC11451052; doi:10.1083/jcb.202311189)
Supplement: Table S10 — shows a localization precision table in relation to Fig. 2 F. [file JCB_202311189_TableS10.docx]

**Table S10. Localization precision table in relation to Fig. 2F**

| **Target** | **Localization precision (nm)** |
| --- | --- |
| GM130 | 18.4 |
| GLANT2 | 16.8 |
| Golgin-97 | 22.1 |
| beta-COP | 19.9 |
| TGN46 | 21.6 |
| Rab6 | 20.3 |
| Giantin | 20.8 |
| GRASP65 | 22.1 |
| FAM177A1 | 20.5 |
| VPS13B | 20.4 |
