# Supplementary material for: VPS13B is localized at the interface between Golgi cisternae and is a functional partner of FAM177A1
Source: J Cell Biol. 2024 Sep 27;223(12):e202311189. doi: 10.1083/jcb.202311189 (PMC11451052; doi:10.1083/jcb.202311189)

Source Data Figure 3B

Immunoblot: VPS13B

Low exposure

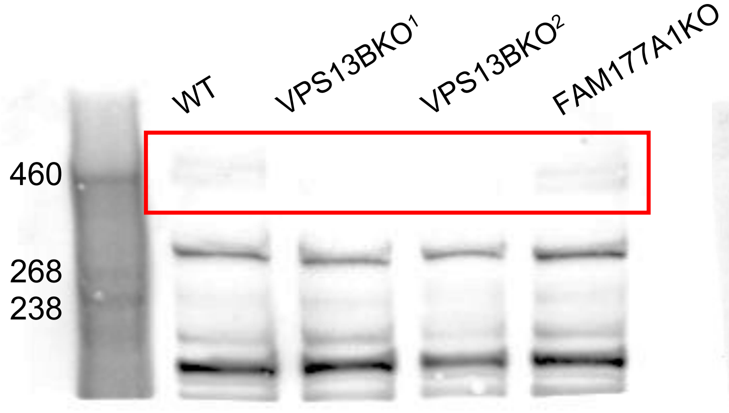

High exposure

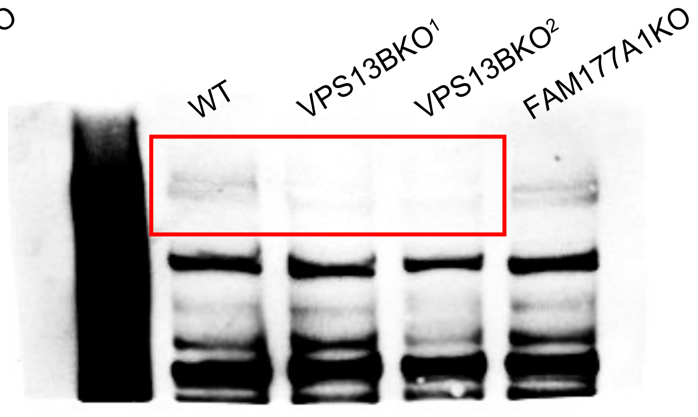

Ladder: HiMark

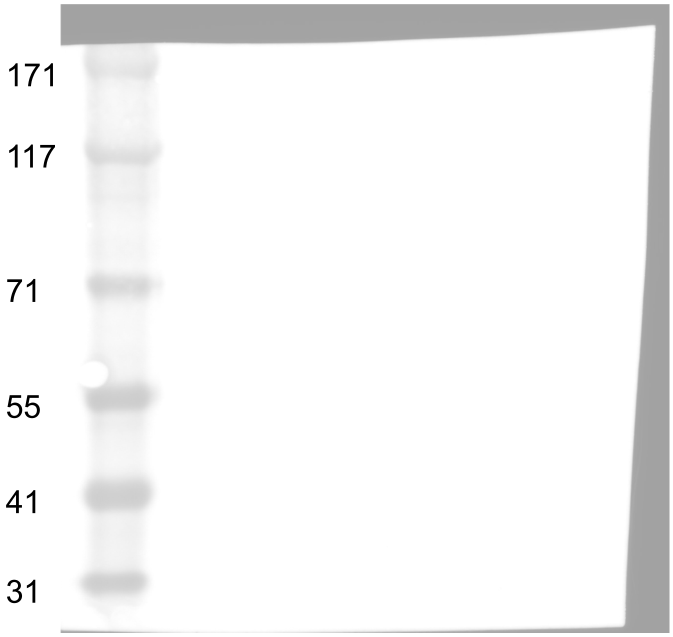

Immunoblot: GAPDH

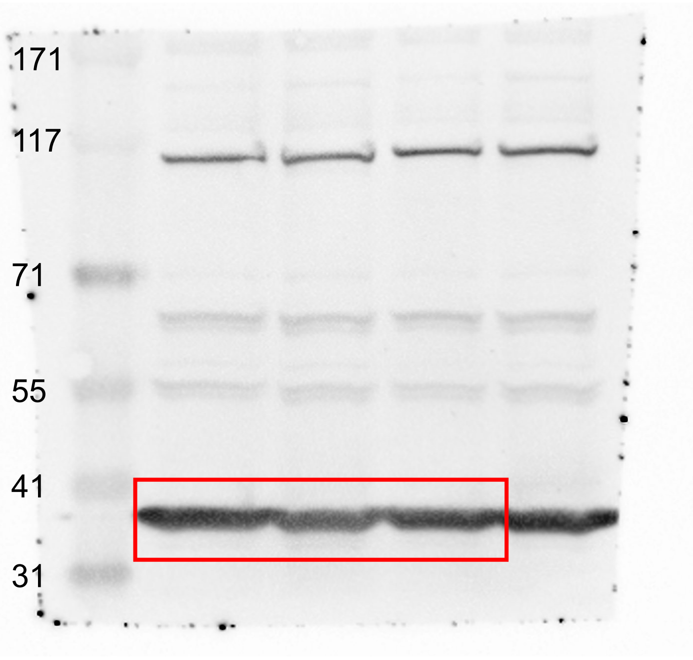

Source Data Figure 3C

Immunoblot: anti-FAM177A1

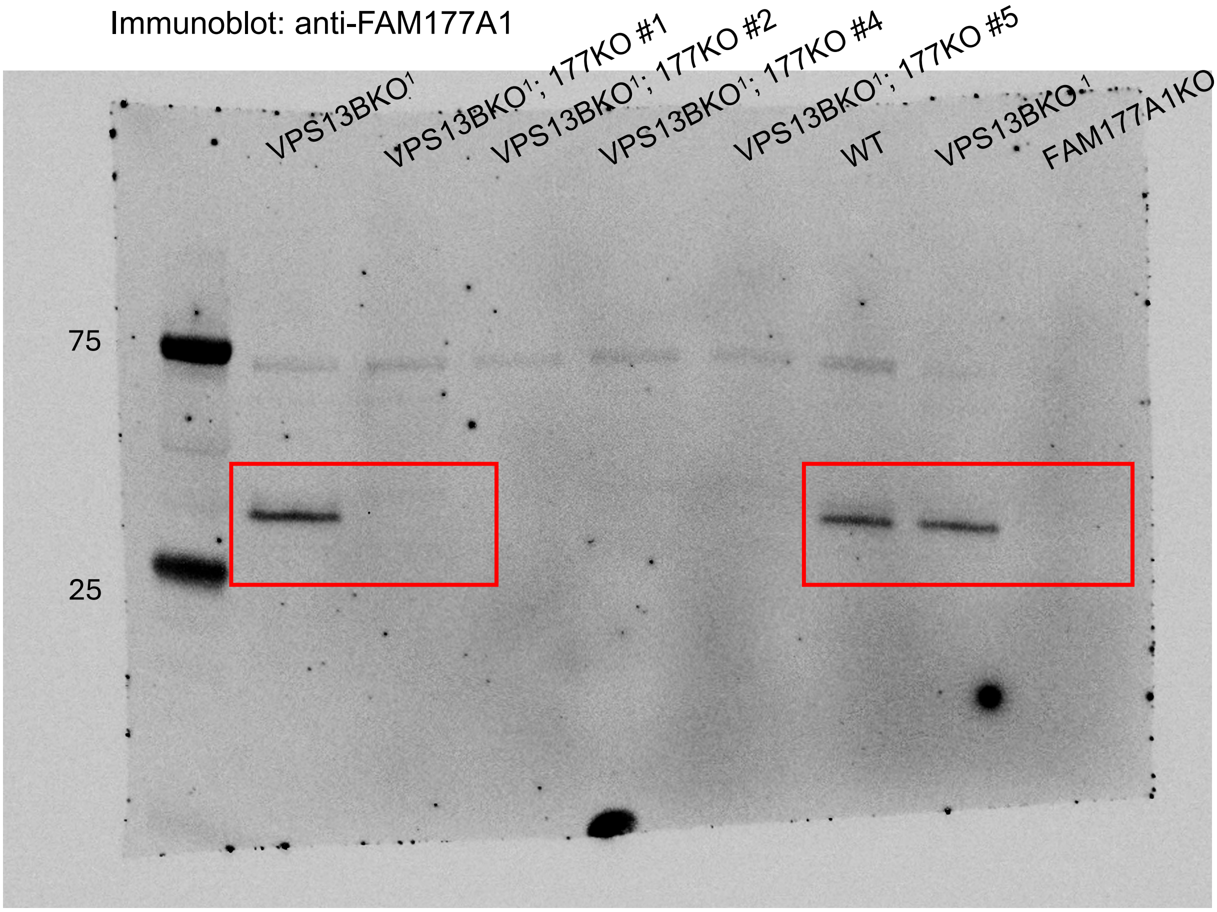

Immunoblot: anti-GAPDH

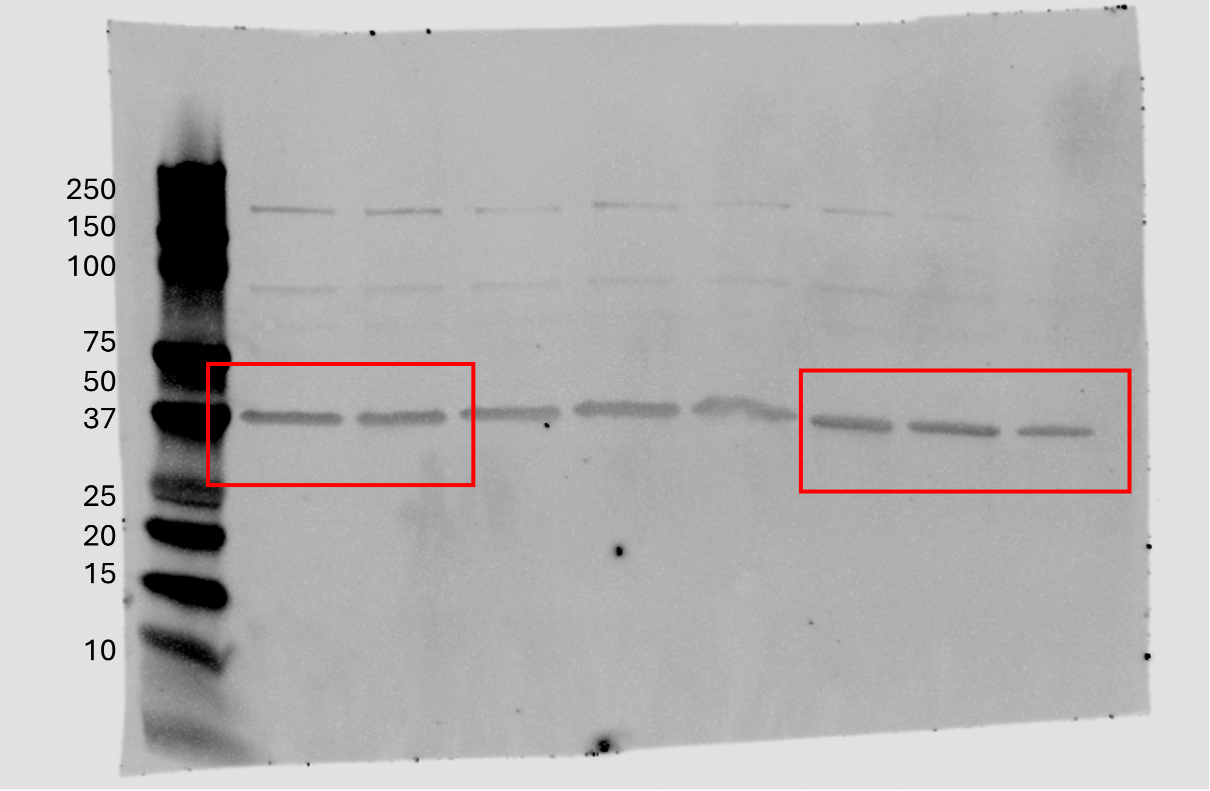

Supplement: SourceData F3 — is the source file for Fig. 3. [file JCB_202311189_SourceDataF3.pdf]
